# Single-center experience with a unibody single-branched stent graft for zone 2 thoracic endovascular aortic repair

**DOI:** 10.3389/fcvm.2022.995173

**Published:** 2022-09-09

**Authors:** Xiang Kong, Peng Ruan, Jiquan Yu, Tianshu Chu, Lei Gao, Hui Jiang, Jianjun Ge

**Affiliations:** Department of Cardiovascular Surgery, The First Affiliated Hospital of USTC, Division of Life Sciences and Medicine, University of Science and Technology of China (USTC), Hefei, China

**Keywords:** thoracic endovascular aortic repair (TEVAR), aortic dissection, aortic arch, thoracic aortic disease, thoracic aortic aneurysm, left subclavian artery, branched stent graft

## Abstract

To provide an adequate proximal landing zone, left subclavian artery (LSA) reconstruction has become an important part of thoracic endovascular aortic repair (TEVAR). This study evaluates the short and medium term efficacy of a novel unibody single-branched stent graft for zone 2 TEVAR. Fifty-two patients (mean age, 56 ± 10.9 years; 42 men) with distal aortic arch lesions requiring LSA reconstruction received unibody single-branched stents from September 2019 to March 2021. Computed tomography angiography was performed 6, 12, and 24 months after surgery to observe stent morphology, branch patency, endoleaks, stent-related adverse events, and changes in the diameter of true and false lumens. All stents were deployed adequately, and the technical success rate was 100%. The mean operation time was 121.8 ± 47.0 min. The mean postoperative hospital stay was 6.2 ± 3.7 days, and the mean follow-up was 16.8 ± 5.2 months (range, 12–24 months). During follow-up, there were no deaths and complications such as stent displacement or fracture, stenosis, fracture, occlusion, and type Ia endoleaks. The patency rate of the branched segment was 100%. In 42 patients with aortic dissection (AD), the true lumen diameter of the aortic isthmus was 29.4 ± 2.9 mm after surgery, significantly larger than before surgery (20.6 ± 5.4 mm, *P* < 0.05). Postoperative aortic isthmus false lumen diameter was significantly smaller than that before operation (6.1 ± 5.2 mm vs. 16.0 ± 7.6 mm, *P* < 0.05). The new unibody single-branched stent for zone 2 TEVAR is safe and accurate, and its efficacy is good in the short and medium term.

## Introduction

With surgical advancements, thoracic endovascular aortic repair (TEVAR) has replaced open surgery for treating thoracic aortic aneurysms and has become the preferred treatment for distal aortic arch disease. However, more than 40% of patients in clinical practice need to extend stent graft to landing zone 2 for adequate endovascular repair (1, 2).

Blood flow to the left subclavian artery (LSA) can be restored using different techniques, including parallel (chimney) stents, fenestration, and carotid-subclavian bypass. Nonetheless, these methods are technically difficult, increasing the risk of endoleaks and neurological complications (3–5). For these patients, single-branched stent grafts provide good anchoring and LSA flow. This study describes the case of 52 patients with distal aortic arch disease treated with a new unibody single-branched stent graft in our center, with good results in the short and medium term.

## Materials and methods

### Patient population

Fifty-two patients with distal aortic arch disease and inadequate proximal landing zone were treated with a unibody single-branched stent graft from September 2019 to March 2021 at the First Affiliated Hospital of the University of Science and Technology of China. This retrospective study was approved by the ethics committee of our institution, and all patients gave written informed consent before operation (IRB number: 2021-RE-148).

The inclusion criteria were (1) men and non-pregnant women aged 18–80 years; (2) diagnosis of distal aortic arch disease, including aortic dissection (AD), penetrating aortic ulcer (PAU), thoracic aortic aneurysm (TAA) with a diameter of >5.5 cm, and rapid aortic growth (>1 cm/year); (3) distance between the proximal end of the aortic lesion and the LSA ostium <15 mm; (4) aortic disease not affecting the left common carotid artery (LCCA), and distance between the proximal end of the aortic lesion and the LCCA ostium >15 mm; (5) distance from the origin of the left vertebral artery (LVA) to the LSA ostium >25 mm; (6) distance between the LCCA ostium and the LSA ostium >5 mm; and (7) aortic arch diameter <40 mm.

The exclusion criteria were (1) connective tissue diseases, including Marfan syndrome and Ehlers-Danlos syndrome; (2) severe organ disease that prevented the execution of surgery or anesthesia; (3) Stanford type A AD; (4) aberrant right subclavian artery; (5) diameter of the external iliac artery or common femoral artery [FA] <7 mm; (6) allergy to nitinol or iodine contrast agents.

All patients underwent high-resolution computed tomography angiography (CTA) of the aorta before operation. CTA data were measured and reconstructed using an Endosize vascular imaging workstation (Endosize; Therenva Inc., Rennes, France). The centerline along the aorta was set up during 3D reconstruction to improve the accuracy of measurements, except in cases in which the length of the aortic arch and the distance between aortic branches were measured along the greater curvature.

The type of aortic disease (AD, TAA, or PAU), the area involved, and the aortic arch shape were evaluated by an experienced surgeon based on the results of 3D reconstruction. The diameter of the proximal and distal landing zone, maximum LSA diameter, diameter of the LSA at 25 mm distal from the ostium, LSA tortuosity and stenosis, distance between the LVA origin and the LSA ostium, distance from the LCCA ostium to the LSA ostium, and the characteristics of common and external iliac artery and FA (diameter, stenosis, calcification, and tortuosity) were also measured. The area of dissection, number and location of intimal tears, distance between the proximal intimal tear and the LSA ostium, relationship of visceral and renal perfusion with the true and false aortic lumen, and diameter of true and false lumens were determined in cases involving AD. The optimal C-arm angle was calculated by volume rendering and centerline reconstruction to facilitate the observation of LSA ostium during surgery and to facilitate the alignment of branch and LSA with the help of a marker band on the graft. Acute type B AD and PAU were treated with medications to control blood pressure and heart rate. After completing relevant examinations on admission, surgery was performed at least 1 week after the onset of acute AD, when the clinical condition was stable.

### Stent graft

**FIGURE 1:**
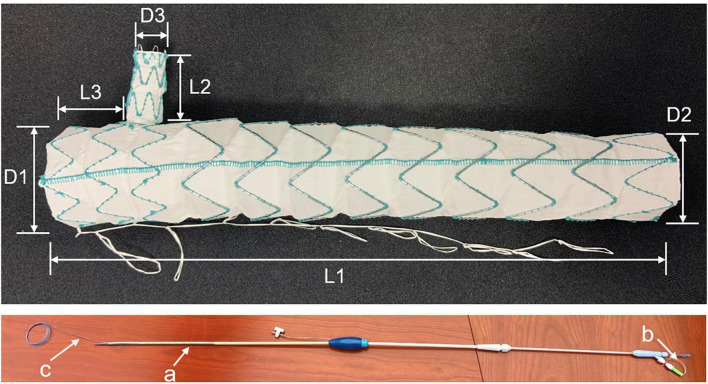
Castor single-branched stent graft and the delivery system. a, 24-Fr outer sheath; b, trigger wire; c, traction wire.

All patients were treated with Castor single-branched stent grafts (MicroPort Endovascular, Shanghai, China). The graft was made of nitinol and polyester and was composed of a main body and a branch. The specifications of the stent graft are shown in Figure 1. The device was deployed using a 24-F delivery sheath. Four parameters were selected based on preoperative CTA measurements: the diameter of the proximal and distal end of the main body, the diameter of the distal end of the branch, and the distance from the ostium of the branch to the proximal end of the main body. Graft oversizing varied depending on the type of aortic disease. The diameter of the proximal end of the graft was oversized by <5% in patients with acute dissection and by 5–15% in patients with chronic dissection, PAU, or TAA. The diameter of the distal end of the graft varied depending on the true lumen diameter and was oversized by <20%.

### Stent graft deployment

All surgeries were performed under general anesthesia. Heparin (100 U/kg) was administered with a target activated coagulation time of 300 s. Visceral perfusion and the location of lesions in the thoracic aorta were assessed by angiography. The contralateral common FA was exposed through a groin incision. The left brachial artery (LBA) was cannulated with a 6-Fr sheath. A 5-Fr MPA1 catheter was advanced over a guidewire to the exposed FA through the sheath.

**FIGURE 2:**
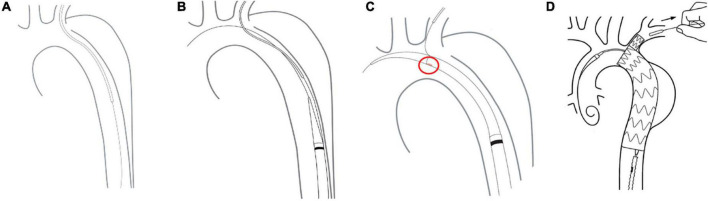
Delivery and deployment of a Castor single-branched stent graft. **(A)** The guidewire is advanced from the femoral artery to the left brachial artery. **(B)** The delivery system is inserted into the descending aorta. **(C)** The correct position of the branch is assessed by the alignment of a marker band. **(D)** Stent deployment.

**FIGURE 3:**
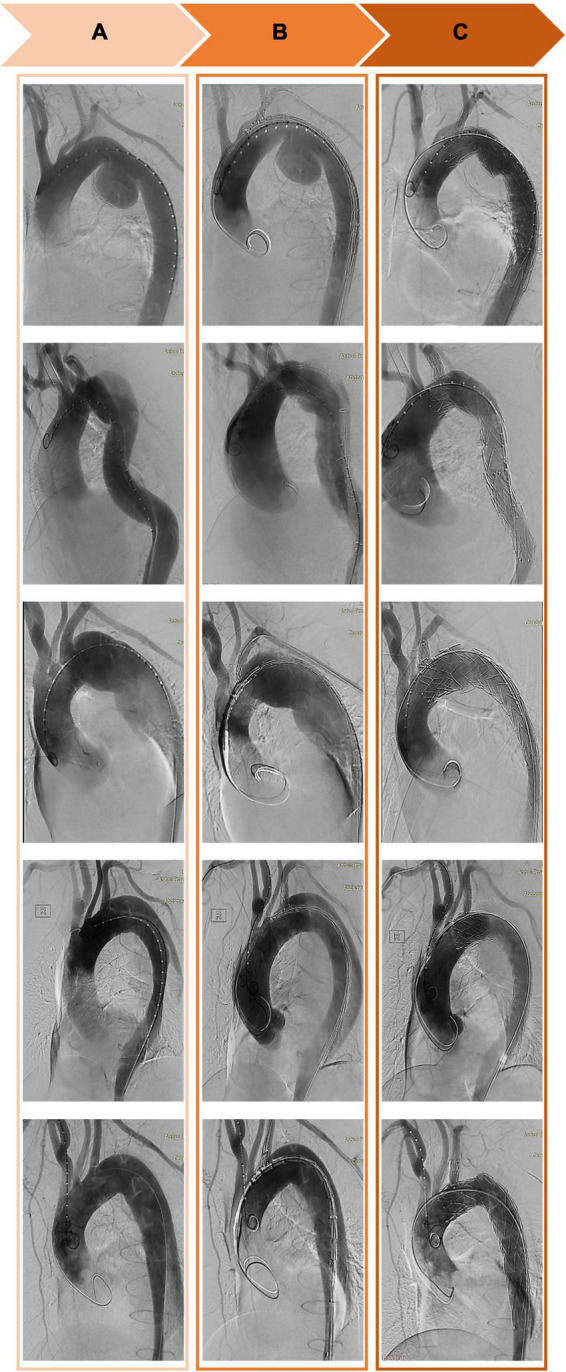
Computed tomography angiography of the thoracic aorta before **(A)**, during **(B)**, and after **(C)** implantation of a Castor single-branch stent graft.

The free end of a traction wire attached to the graft branch was inserted into the MPA1 catheter through the FA and out of the LBA. A super stiff guidewire (Lunderquist; Cook, Bloomington, IN, United States) was introduced into the ascending aorta through the same femoral route. The delivery sheath was introduced along the super stiff guidewire until it reached the descending aorta. The delivery system was rotated until the branch was located at the greater curvature of the aortic arch. The stent and the soft inner sheath were advanced into the aortic arch. We checked whether the position of the branch was correct or the branch traction wire was twisted. The soft sheath was withdrawn, and the branch was pulled into the LSA using the traction wire. To deploy the main body, systolic blood pressure would be maintained at about 90 mmHg by pharmacological therapy. After locating the stent under X-ray, the trigger wire was pulled to deploy the main body. Then, the traction wire was pulled to deploy the branch (Figure 2). Shortly after deployment, normal blood pressure was restored and angiography was performed to evaluate aortic lesion isolation and branch patency. The delivery system and guidewires were removed (Figure 3). The FA was sutured, and LBA was compressed for 20 min before bandaging. After awakening from anesthesia, the patient returned to the ward for clinical monitoring. After surgery, the patient was treated with aspirin 100 mg once a day for 1 year.

### Follow-up

All patients underwent physical examination 30 days after surgery. Aortic CTA was performed 6, 12, and 24 months postoperatively. Our patients were followed up until April 2022, and no patients were lost to follow-up. False lumen thrombosis and aortic remodeling in AD patients were evaluated by CTA at the last follow-up. Aortic remodeling was assessed at the aortic isthmus, pulmonary artery bifurcation, and diaphragm levels. A straight line bisecting the center of the intimal flap and perpendicular to the centerline of blood flow was considered the diameter of the true and false lumens at the cross-sectional plane.

### Statistical analysis

Statistical analysis was performed using SPSS version 22.0 (SPSS Inc., Chicago, IL, United States). Continuous variables were presented as mean ± SD, and categorical data were expressed as count (percentage). Changes in the diameter of true and false lumens between before and after surgery were assessed using a paired *t*-test. A *p*-value of less than 0.05 was considered statistically significant.

## Results

### Baseline characteristics

The mean age of our cohort (42 males and ten females) was 56 ± 10.9 years (range, 29–78 years). The diseases in our study included acute AD (time from onset to TEVAR <2 weeks, 32 cases), chronic AD (>2 weeks, 10 cases), penetrating ulcer complicated by hematoma (six cases), and aortic aneurysm (four cases). No patients had connective tissue diseases, microbial infections, or other surgical complications. Moreover, there were 45 cases (86.5%) of hypertension, 11 cases (21.2%) of coronary heart disease, and six cases (11.5%) of diabetes (Table 1).

**TABLE 1:** Patient characteristics before stent graft implantation for the endovascular repair of the thoracic aorta.

Variables	*n* = 52
Age, years	56 ± 10.9
**Sex**	
Female	10 (19.2%)
Male	42 (80.8%)
Diabetes	6 (11.5%)
Hypertension	45 (86.5%)
Chronic obstructive pulmonary disease	4 (7.7%)
Peripheral artery disease	2 (3.8%)
Heart failure	5 (9.6%)
Dialysis dependence	1 (1.9%)
Prior stroke	3 (5.8%)
Previous endovascular aortic aneurysm repair	0 (0%)
Coronary heart disease	11 (21.2%)
Prior myocardial infarction	2 (3.8%)
Previous coronary artery bypass grafting	0 (0%)
Carotid stenosis	1 (1.9%)
Smoker	24 (46.2%)
**Etiology**	
Acute type B	32 (61.5%)
Chronic type B	10 (19.2%)
Penetrating aortic ulcer	6 (11.5%)
Aneurysm	4 (7.7%)

The results are mean ± standard deviation.

### Perioperative outcomes

Preoperative CTA showed that the distance between the proximal end of the lesion and the LSA ostium was less than 15 mm in all patients, with an average of 8.4 ± 4.2 mm. The distance from the proximal end of the lesion to the LCCA ostium was 26.0 ± 5.3 mm. The distance between the LCCA ostium and the LSA ostium was 12.6 ± 4.0 mm. The diameter of the proximal landing zone was 30.8 ± 3.0 mm. The average diameter of the proximal end of the stent graft was 32.9 ± 2.7 mm, and the proximal end of the stent was oversized by 7.4% ± 4.9%. The average diameter of the LSA at 25 mm distal from the ostium was 9.9 ± 1.3 mm, and the average diameter of the distal end of the branch was 10.4 ± 1.9 mm. The mean distance between the branch and the proximal end of the main body was 9.7 ± 3.0 mm. There were 18 cases of type I aortic arch, 21 cases of type II aortic arch (including two cases of bovine arch), 13 cases of type III aortic arch (including one case of bovine arch), and one case of LVA originating from the aortic arch (Table 2).

**TABLE 2:** Operative characteristics and outcomes at 30 days.

Variables	*n* = 52
**Type of arch (Myla)**	
I	18 (34.6%)
II	21 (40.4%)
III	13 (25.0%)
Distance from the proximal end of the aortic lesion to the LSA ostium (mm)	8.4 ± 4.2
Distance from the proximal end of the aortic lesion to the LCCA ostium (mm)	26.0 ± 5.3
Distance between the LCCA ostium and the LSA ostium (mm)	12.6 ± 4.0
Proximal aortic diameter (mm)	30.8 ± 3.0
Proximal stent graft diameter (mm)	32.9 ± 2.7
Oversize rate of the proximal aortic landing zone	0.074 ± 0.049
Distal LSA diameter (mm)	9.9 ± 1.3
Diameter of the distal end of the branch (mm)	10.4 ± 1.9
Distance from the proximal end of the main body and the branch (mm)	9.7 ± 3.0
Technical success rate (%)	100
Operation time (min)	124 ± 44.0
Fluoroscopy time (min)	27.2 ± 10.0
Contrast agent volume (ml)	119 ± 21.0
Hospital stay after the operation (days)	6.2 ± 3.7
Minor stroke	2 (3.8%)
Endoleak	5 (9.6%)
30-day mortality	0 (0.0%)
Retrograde type A dissection	0 (0.0%)
Major complications in hospital	0 (0.0%)

LSA, left subclavian artery; LCCA, left common carotid artery.

**FIGURE 4:**
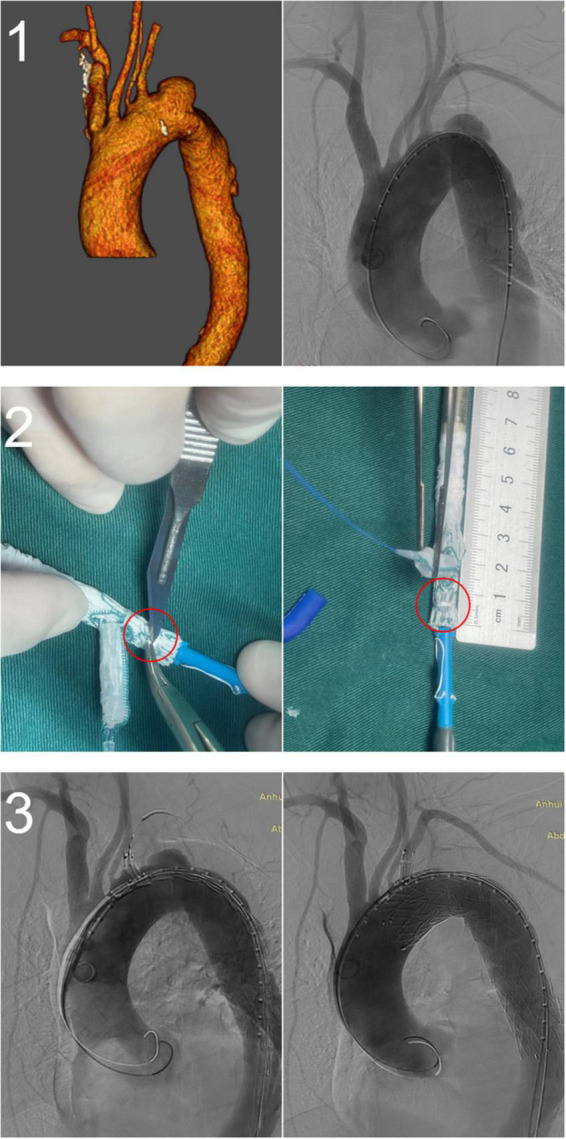
A fenestrated-branched stent graft was used to reconstruct the left subclavian artery (LSA) and the left vertebral artery (LVA). (1) Preoperative computed tomography angiography showing that the LVA originated from the aortic arch. (2) *In vitro* fenestration of the Castor stent graft. (3) Intraoperative and postoperative angiography showing the successful reconstruction of the LSA and LVA.

All stents were successfully deployed without changes in shape, and lesions in the distal aortic arch were treated satisfactorily. There were no cases of type Ia endoleak, complications requiring surgical treatment, or intraoperative death. One patient was treated with a restrictive stent because the distal end of the stent graft was oversized >20%. The left axillary artery was accessed through an additional infraclavicular incision in one patient because of difficulty puncturing the LBA. One patient underwent *in vitro* fenestration because the LVA originated from the aortic arch (Figure 4). The mean operation time was 124 ± 44 min, fluoroscopy time was 27.2 ± 10.0 min, and the contrast agent volume was 118.6 ± 20.8 ml. In all cases, immediate postoperative angiography showed that the lesions were treated successfully, LSA perfusion was good, and the technical success rate was 100%. There were no deaths and no cases of type II or type III endoleaks, spinal cord injury, left upper limb ischemia, contrast-induced nephropathy, or other serious complications 30 days after the operation. All patients were discharged after surgery, and the mean length of postoperative stay was 6.2 ± 3.7 days. During the perioperative period, two patients (3.8%) had a minor stroke, but none had a major stroke. After treatment, the patients recovered completely before discharge (Table 2).

### Follow-up

Postoperative follow-up was 12–24 months, with an average of 16.8 ± 5.2 months. None of our patients died during follow-up. Aortic CTA was reviewed at 6, 12, and 24 months after surgery. The aorta and LSA were unobstructed, and the patency rate was 100%. CTA showed that intimal tear in the distal descending aorta persisted, and the false lumen in the descending aorta was enlarged in one patient at 12 months after surgery. The tear was treated by stent implantation, and the patient recovered well. All the other patients survived during follow-up without stent displacement, branch occlusion, left upper limb ischemia, open surgical repair, aortic rupture, stent graft-induced new distal tear, retrograde type A AD, stroke or paraplegia, and other surgical complications.

**FIGURE 5:**
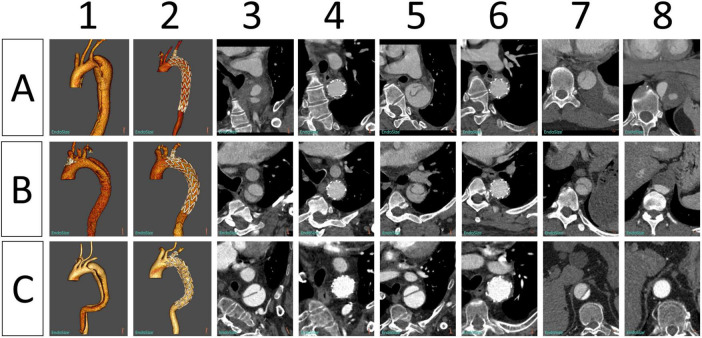
Morphological changes in the aorta in three patients **(A–C)** before and after implantation of Castor single-branched stent grafts. (1, 2) Preoperative and postoperative 3D reconstruction of the thoracic aorta. (3, 4) Preoperative and postoperative images of the aortic isthmus. (5, 6) Preoperative and postoperative images at the level of the pulmonary artery bifurcation. (7, 8) Preoperative and postoperative images of the aorta at the level of the diaphragm.

In 42 patients with AD, the last CTA examination showed complete thrombosis in the false lumen of distal aortic arch and proximal descending aorta. The rate of complete thrombosis in the aortic isthmus was 100%. Complete thrombosis in the false lumen at the level of the pulmonary artery bifurcation occurred in 39 patients, and partial thrombosis was observed in three patients. Complete and partial thrombosis at the level of the diaphragm occurred in 19 and 23 patients, respectively (Table 3). The false lumen diameter at the level of the aortic isthmus and pulmonary artery bifurcation decreased significantly (*P* < 0.01), whereas the true lumen diameter increased significantly (*P* < 0.01). Thrombosis in the distal aorta was partial because of the presence of re-entry tears. There were no significant differences in the diameter of true and false lumens at the diaphragm level between before and after the operation (*P* > 0.05) (Table 4 and Figure 5).

**TABLE 3:** False lumen thrombosis in 42 patients with aortic dissection.

Level	*n* = 42
**Aortic isthmus**	
CT	42 (100%)
PT	0 (0%)
Pulmonary artery bifurcation	8.4 ± 4.2
CT	39 (92.9%)
PT	3 (7.1%)
**Diaphragm**	
CT	19 (45.2%)
PT	23 (54.8%)

CT, complete thrombosis; PT, partial thrombosis.

**TABLE 4:** Changes in aortic diameter in 42 patients with aortic dissection.

Level	Before graft placement (mm)	After graft placement (mm)	*P*-value
**Aortic isthmus**			
True lumen	20.6 ± 5.4	29.4 ± 2.9	<0.01
False lumen	16.0 ± 7.6	6.1 ± 5.2	<0.01
**Pulmonary artery bifurcation**			
True lumen	17.9 ± 6.3	26.7 ± 3.9	<0.01
False lumen	19.3 ± 8.5	10.8 ± 8.6	<0.01
Diaphragm			
True lumen	15.3 ± 6.4	16.4 ± 6.7	0.062
False lumen	14.5 ± 7.1	13.6 ± 8.3	0.168

The results are mean ± standard deviation.

## Discussion

TEVAR has become the mainstream treatment for descending aortic disease (6, 7). However, the presence of limited proximal landing zone (i.e., distance from lesion to the LSA >15 mm) in some patients increased surgical difficulty and the incidence of perioperative complications, such as bird-beak configuration, type Ia endoleaks, and new retrograde dissection into the aortic arch and ascending aorta (8). Therefore, zone 2 must be covered to avoid these complications. Intraoperative coverage of the LSA is the simplest way to extend the proximal landing zone to the LCCA ostium. However, complete coverage of the LSA may lead to left upper limb ischemia, subclavian artery steal syndrome, and vertebral artery cerebral ischemia, increasing the risk of paraplegia (1, 9, 10). Therefore, recent guidelines suggest performing LSA revascularization in elective surgery (6). However, there is controversy regarding whether the best strategy is hybrid, chimney, fenestrated, or branched technique. The hybrid technique combines the traditional LCCA-LSA bypass and TEVAR to extend the proximal landing zone through conventional surgery. This method is simple and reliable but increases the risk of hematoma, chylous leakage, and nerve injury (11–13). Total endovascular techniques, including chimney, fenestration and branched endograft technique reduce surgical risks. The chimney technique is a relatively simple method used for LSA reconstruction. However, the gutter between the chimney stent and the main stent increases the risk of type Ia endoleaks, and the constant pressure of the main stent may lead the occlusion of the branch stent; thus, this approach should be used with caution (3, 14, 15). In *in vitro* fenestration, it is more difficult and risky to align the fenestrated graft to the LSA ostium because of the curvature of the aortic arch (5, 16–18). *In situ* fenestration requires several pieces of equipment, including laser, wires, catheters, and balloons, and may decrease stent strength, increasing the risk of cerebral ischemia. In addition, this method is technically difficult and time-consuming. Graft tears may lead to type III endoleaks, which are difficult to treat (19, 20).

Branched endografts have been developed in recent years. These endografts are associated with a lower risk of endoleaks and are better accommodated in the aortic arch curvature. Moreover, they do not cause type Ia endoleaks (common in chimney grafts) and type III endoleaks (common in *in situ* fenestration), thus being ideal for treating distal aortic arch lesions with limited proximal landing zone (21, 22).

The use of unibody branched endografts for treating distal aortic arch aneurysms involving the LSA was first reported by Inoue et al. However, the incidence of complications using this method, such as endoleaks and cerebral infarction, is high (23, 24). Almost all single-branched and multi-branched grafts are currently in early clinical trials with good results but are not yet commercially available. Single-branched devices include thoracic branch endoprosthesis (TBE) (W.L. GORE, Flagstaff, AZ, United States), Valiant Mona LSA (Medtronic, Santa Rosa, CA, United States), and Nexus (Endospan, Herzelia, Israel). TBE (25) and Mona LSA (26, 27) are used to reconstruct the LSA, and the anchoring area was extended to zone 2. Nexus is deployed on zone 0 and is used to reconstruct the brachiocephalic trunk (28). Multi-branched devices include the Double Branch Relay (Terumo Aortic, Ft. Lauderdale, FL, United States) (29, 30) and two- and three-branched grafts (Cook Medical, Bloomington, IN, United States) (31–33). The brachiocephalic trunk, LCCA, and LSA can be reconstructed simultaneously, extending the anchoring area to zone 0. The Castor stent is the only branched endograft currently available in China (34, 35). Stents with different sizes and deployment sites are designed according to vessel anatomy to standardize treatment and increase the stability between the main body and branches. To minimize the risk of injuries, stents poorly positioned in the arch can be retracted into the descending aortic sheath and rotated, reducing aortic arch manipulation and aortic wall damage. In addition, the soft inner sheath reduces the risk of intimal damage and cerebral embolism.

The fact that none of our patients had a major stroke may be due to stent design, young age (56 ± 10.9 years), pathological characteristics (no obvious atherosclerotic plaques in the arch), and the small sample size. There were no type Ia endoleaks probably because the landing zone was more than 20 mm, the diameter of the proximal aorta was less than 40 mm, and the sample size was small.

Our results show that the unibody branched endograft is safe and effective. However, the device has some shortcomings. First, the relatively large diameter (24F) of the outer sheath limited the application of the delivery system in patients with small FA. Second, all stents were 200 mm in length and 6 mm in taper, limiting their use in patients with large or small differences in diameter between the proximal and distal landing zones. Third, surgery was relatively complex. Fourth, the traction wire might become entangled with the super stiff guidewire, causing serious problems during deployment. Notwithstanding, the last two problems can be solved by clinical training.

## Conclusion

For distal aortic arch lesions with inadequate proximal landing zone, Castor single-branched stent grafts are easily deployed, safe, and provide effective endovascular treatment of the thoracic aorta in the short and medium terms. Nonetheless, these stents can be improved further, and larger multicenter controlled clinical trials with longer follow-up are needed to assess the long-term effects of these stents.

## Data availability statement

The original contributions presented in this study are included in the article/supplementary material, further inquiries can be directed to the corresponding authors.

## Ethics statement

The studies involving human participants were reviewed and approved by The First Affiliated Hospital of University of Science and Technology of China. Written informed consent for participation was not required for this study in accordance with the national legislation and the institutional requirements.

## Author contributions

XK and JG: conception and design, provision of study materials or patients, and administrative support. XK, PR, and JY: collection and assembly of data. XK, TC, LG, and HJ: data analysis and interpretation. All authors: manuscript writing. All authors read and approved the final manuscript.
